# Sickness at Algiers

**Published:** 1847-01

**Authors:** 


*Sickness at Algiers.*—According to the Gazette Mèdicale, the hospitals of Algiers, are at present overcrowded with the sick. Numbers of patients labouring under fever are daily rejected from the civil hospital. Notwithstanding the exertions of the medical officers, numerous patients die without assistance.—*Med. Gaz.*

